# The Pfs230 N-terminal fragment, Pfs230D1+: expression and characterization of a potential malaria transmission-blocking vaccine candidate

**DOI:** 10.1186/s12936-019-2989-2

**Published:** 2019-11-08

**Authors:** Shwu-Maan Lee, Yimin Wu, John M. Hickey, Kazutoyo Miura, Neal Whitaker, Sangeeta B. Joshi, David B. Volkin, C. Richter King, Jordan Plieskatt

**Affiliations:** 1 PATH’s Malaria Vaccine Initiative (MVI), 455 Massachusetts Avenue NW, Suite 1000, Washington, DC 20001-2621 USA; 2 Department of Pharmaceutical Chemistry, Vaccine Analytics and Formulation Center, University of Kansas, Lawrence, KS 66047 USA; 3 Laboratory of Malaria and Vector Research, National Institute of Allergy and Infectious Diseases, National Institutes of Health, Rockville, MD 20852 USA

**Keywords:** Malaria, Pfs230, *Plasmodium falciparum*, Transmission-blocking vaccine, Baculovirus, Glycosylation, Recombinant protein

## Abstract

**Background:**

Control and elimination of malaria can be accelerated by transmission-blocking interventions such as vaccines. A surface antigen of *Plasmodium falciparum* gametocytes, Pfs230, is a leading vaccine target antigen, and has recently progressed to experimental clinical trials. To support vaccine product development, an N-terminal Pfs230 antigen was designed to increase yield, as well as to improve antigen quality, integrity, and homogeneity.

**Methods:**

A scalable baculovirus expression system was used to express the Pfs230D1+ construct (aa 552–731), which was subsequently purified and analysed. Pfs230D1+ was designed to avoid glycosylation and protease digestion, thereby potentially increasing homogeneity and stability. The resulting Pfs230D1+ protein was compared to a previous iteration of the Pfs230 N-terminal domain, Pfs230C1 (aa 443–731), through physiochemical characterization and in vivo analysis. The induction of functional antibody responses was confirmed via the standard membrane feeding assay (SMFA).

**Results:**

Pfs230D1+ was produced and purified to an overall yield of 23 mg/L culture supernatant, a twofold yield increase over Pfs230C1. The Pfs230D1+ protein migrated as a single band via SDS-PAGE and was detected by anti-Pfs230C1 monoclonal antibodies. Evaluation by SDS-PAGE, chromatography (size-exclusion and reversed phase) and capillary isoelectric focusing demonstrated the molecule had improved homogeneity in terms of size, conformation, and charge. Intact mass spectrometry confirmed its molecular weight and that it was free of glycosylation, a key difference to the prior Pfs230C1 protein. The correct formation of the two intramolecular disulfide bonds was initially inferred by binding of a conformation specific monoclonal antibody and directly confirmed by LC/MS and peptide mapping. When injected into mice the Pfs230D1+ protein elicited antibodies that demonstrated transmission-reducing activity, via SMFA, comparable to Pfs230C1.

**Conclusion:**

By elimination of an *O*-glycosylation site, a potential *N*-glycosylation site, and two proteolytic cleavage sites, an improved N-terminal Pfs230 fragment was produced, termed D1+, which is non-glycosylated, homogeneous, and biologically active. An intact protein at higher yield than that previously observed for the Pfs230C1 fragment was achieved. The results indicate that Pfs230D1+ protein produced in the baculovirus expression system is an attractive antigen for transmission-blocking vaccine development.

## Background

Transmission-blocking vaccines (TBVs) for malaria are designed to interrupt parasite transmission [1–3] and likely to be preferentially deployed in combination with other antigens targeting other life cycle stages [4, 5]. In human hosts, TBVs induce antibodies against sexual stage malaria antigens or mosquito antigens, thereby blocking the parasite fertilization after an *Anopheles* mosquito takes a blood meal from a parasite-infected individual, and thus breaking the cycle of parasite transmission between human and mosquito hosts [1–3]. The expression and purification of the Pfs230 protein, a leading TBV candidate, has been challenging due to the large size and complexity of the protein, which is rich in disulfide bonds and contains multiple domains [6]. However, N-terminal fragments of Pfs230 have been successfully expressed in *Pichia pastoris* [7], in wheat germ cell free lysates [8], in plant [9] and in baculovirus [10]. The most clinically advanced Pfs230 candidate (Pfs230 D1M) is produced in *Pichia* [7] and chemically conjugated to *Pseudomonas aeruginosa* exoprotein A (EPA), a carrier protein demonstrated to enhance immunogenicity of the target antigen [11, 12]. Clinical trials are underway to test Pfs230-EPA in combination with GSK’s AS01 adjuvant [13] and initial results have been promising [ClinicalTrail.gov Identifier: NCT02942277].

With the strong rationale and development data on Pfs230-based vaccines, it is important to develop constructs of Pfs230 that are pure and have potential for commercial development. The successful production of an N-terminal fragment of Pfs230 in the baculovirus expression system using super Sf9 cells was previously reported [10], and lessons learned from these previous studies was utilized to accelerate the development of a second-generation N-terminal Pfs230 TBV candidate. The prior protein, Pfs230C1 (aa 443–731), was characterized to be monomeric with both disulfide bonds properly paired and immunization of mice resulted in the induction of antibodies exhibiting transmission-reducing activity [10]. The production yield of Pfs230C1, however, was only moderate despite efforts in process optimization (~ 10 mg/L fermentation supernatant), and the sub-optimal yield partially attributed to proteolytic degradation and presence of cleaved forms of Pfs230C1 [14]. Additionally, while the glycosylation was consistent between batches, the presence of a glycosylated form complicated the recombinant protein characterization [14] and overall product quality. Moreover, since the native parasite surface proteins completely lacked N- or O-glycosylation [15], any glycosylated forms of the recombinant protein do not mimic the natural target and thus are likely undesirable as immunogens.

In the current study, an improved Pfs230 TBV candidate, Pfs230D1+, was investigated in the baculovirus expression system by altering the starting amino acid (aa 552) to avoid glycosylation and potential proteolytic sites. The improvement in antigen design eliminated the undesirable glycosylation as well as resulting in a twofold increase in yield and increased stability. These design iterations are part of a process to optimize the preclinical and clinical development of Pfs230-based vaccines with the goal of a highly effective, low cost, easily deployed vaccine that blocks malaria parasite transmission.

## Methods

### Baculovirus expression construct (Pfs230D1+)

The N-terminal sequence (aa 552–731) of the gametocyte surface protein Pfs230 of 3D7 strain (ACCESSION P68874), containing four cysteines as part of a predicted cysteine-rich domain, was cloned and denoted as Pfs230D1+. Codon optimization for baculovirus expression was performed by DNA2.0 (now ATUM). Synthetic deoxynucleic acid (DNA) of Pfs230D1+ (552–731) contained a N585Q mutation to remove a potential *N*-glycosylation site, an N-terminal secretion signal (MKFLVNVALVFMVVYISYIYAD from Honeybee Melittin) and a C-terminal six histidine tag. Resulting plasmid was cloned, sequence verified, and recombinant bacmids generated as previously described [10]. This bacmid was sequence verified again and used to transfect super Sf9 cells (Oxford Expression Technologies) for the generation of recombinant baculovirus stock using Cellfectin^®^ II reagent (Invitrogen) following Bac-to-Bac manual.

### Expression and purification of Pfs230D1+

Super Sf9 cells were seeded at 1 × 10^6^ cells/mL in SFM4 medium (Hyclone). MOI (multiplicity of infection) of one was used to infect a 10 L super Sf9 wave culture. At 96 h post infection, culture was harvested, concentrated fivefold and diafiltered with 20 mM sodium phosphate, 150 mM NaCl, pH 7.4 (Buffer A) using a tangential flow filtration device (Centrasette LV, Pall) with 0.5 m^2^ Omega polyethersulfone membrane (Pall). Clarification was carried out with 0.22 μm filtration (Stericup-GP vacuum filter, Merck Millipore).

Two litres of clarified, concentrated supernatant was loaded onto a 61 mL (2.6 × 11.5 cm) nickel–nitrilotriacetic acid (Ni–NTA) (His60 Ni Superflow, Clontech) column at 100 cm/h. The wash steps were performed with five column volume (CV) of Buffer A, five CV of buffer A with 10 mM imidazole, then five CV of Buffer A with 20 mM imidazole. The protein was eluted with Buffer A containing 50–100 mM imidazole. Pooled eluents from Ni-NTA column were concentrated fivefold with Amicon Ultra-15 centrifugal filters, using 3 K regenerated cellulose membrane (Merck Millipore). The protein was further purified, and buffer exchanged with size exclusion chromatography (SEC) on a Superdex 75 (2.6 × 60 cm, 320 mL), equilibrated with 20 mM HEPES, 150 mM NaCl, 5% glycerol, pH 7.2. To maintain a 5% CV injection load limit, multiple cycles were performed. The Superdex 75 elution fractions were evaluated by sodium dodecyl sulfate polyacrylamide gel electrophoresis (SDS-PAGE), pooled, and further concentrated to a target of 1 mg/mL using the same Amicon 3 K concentrator (Merck Millipore) and stored at − 80 °C.

### Protein concentration determination

The bicinchoninic acid (BCA) assay was performed according to manufacturer’s instruction (Thermo) to determine protein concentration of purified Pfs230D1+ due to low tryptophan content. Bovine serum albumin was used to generate the protein concentration standard curve in the assay.

### SDS-PAGE

Samples were diluted with 4× LDS (Lithium dodecyl sulfate, Invitrogen) sample buffer, heated for 5 min at 90 °C and loaded in a final volume of 20 μL/well on SDS-PAGE gels (4–12% NuPAGE Bis–Tris, Invitrogen). Gels were run at 150–200 V for 35–50 min in 1× 2-(*N*-morpholino)ethanesulfonic acid (MES) sodium dodecyl sulfate (SDS) running buffer and stained with SimplyBlue™ SafeStain (Invitrogen).

### Western blotting (Anti-His) with purified Pfs230D1+

Following SDS-PAGE, proteins were transferred onto nitrocellulose membrane and Western blot procedure using anti-penta His antibody (Qiagen) as described earlier [10]. The membrane was developed using ECL Prime (GE Healthcare).

### Western blotting using mouse anti-Pfs230C1 monoclonal antibody

Mouse monoclonal antibody 15A4-1B12 was used to evaluate Pfs230D1+ (20 ng) via Western blot as described previously [14].

### Capillary isoelectric focusing (cIEF)

Pfs230D1+ was incubated in either water (non-reduced) or reduced with 10 mM dithiothreitol (DTT) for 30 min at 60 °C, centrifuged for 5 min at 14,000×*g*, and then subjected to cIEF using iCE280 analyzer (Convergent Bioscience). Samples were run in triplicate at 4 °C using a temperature-controlled autosampler. The Pfs230D1+ samples (final concentration of 50 µg/mL) were mixed with Pharmalyte^®^ 3.0–10.0 (GE Healthcare, final concentration of 4%), acidic and basic isoelectric point (pI) markers of 3.6 and 9.5 (Protein-Simple), 0.1% tetramethylethylenediamine, and methyl cellulose (final concentration of 0.35%; Protein-Simple). The samples were separated in 2 focusing periods, one at 1500 V for 1 min and a second at 3000 V for 5 min.

### Size exclusion-ultra high performance liquid chromatography (SE-UHPLC)

Size-exclusion-UHPLC analysis of purified Pfs230D1+ was performed in triplicate on a TSK-Gel BioAssist G25Wxl column (7.8 × 300 mm, TOSOH Biosciences, King of Prussia, PA) at 30 °C on a Shimadzu Prominence UFLC HPLC system with a diode array detector. The mobile phase consisted of 20 mM HEPES, 150 mM NaCl, pH 7.2 with flow rate of 0.7 mL/min. A gel filtration standard (Bio-Rad, Hercules, CA) was used to ensure column and HPLC system integrity prior to each set of runs. The Pfs230D1+ samples were also run with and without the column to determine sample recovery (74 ± 1% for triplicate determinations).

### Reversed-phase (RP) UHPLC

Samples of Pfs230D1+ were incubated in water (non-reduced) or 10 mM DTT (reduced) for 30 min at 60 °C and analysed using an Ultimate 3000 UHPLC system (Thermo Scientific) with an Accucore C4 column (2.6 µm, 2.1 × 150 mm, Thermo Scientific) at a flow rate of 0.2 mL/min. C4 column and auto-sampler temperatures were set at 60 °C and 5 °C, respectively. The elution of Pfs230D1+ was monitored at absorbance (214 nm) using a gradient that consisted of mobile phase (A): water with 0.1% trifluoroacetic acid (TFA) and mobile phase (B): acetonitrile (ACN) with 0.1% TFA as follows: 5 min at 1%B, 10 min 1–70%B, 1 min 70–99%B, 1 min at 99%B, 1 min 99–1%B, and 2 min at 1%B. The non-reduced Pfs230D1+ samples were also run with and without column to determine sample recovery (94 ± 7% for triplicate determinations). The recovery of the reduced sample could not be examined due to interference from DTT.

### Intact mass spectrometry

Pfs230D1+ was incubated in water (non-reduced) or 10 mM DTT (reduced) for 30 min at 60 °C and desalted via a RP BEH C4 trap column (2.1 × 50 mm, Waters, 1.7 µm particles) using Agilent 1220 chromatography system. The intact mass of the protein (in triplicate experiments) was measured using a G6230B time of-flight mass Spectrometer (Agilent Technologies). Mass spectra^1^ (MS^1^) were acquired over a mass range of 400–3200 m/z, with a scan rate of 1 spectra/s. Protein deconvolution was performed using MassHunter (Agilent Technologies).

### LC/MS peptide mapping for N-terminal and disulfide bond analysis

Pfs230D1+ was incubated in water (non-reduced) or 10 mM DTT (reduced) for 30 min at 60 °C, alkylated with 20 mM iodoacetamide and then digested overnight at 37 °C with chymotrypsin. The digested peptides were then subjected to LC/MS using a C18 column (2.1 × 150 mm, 1.7 µm, Thermo Fisher Scientific) operated at 50 °C on UltiMate 3000 UHPL (Thermo Fisher Scientific). The mobile phases consisted of A (water + 0.05% TFA) and B (ACN + 0.05% TFA) and the peptides were eluted using a 5–25% B gradient over 55 min at flow rate of 0.2 mL/min. Peptides were identified using a LTQ-XL ion-trap mass spectrometer (Thermo Fisher Scientific) in positive-ion mode and a mass range of 400–1900 m/z was used. Peptides and disulfide bonds were identified using MS^1^ and MS^2^ data and PepFinder (Thermo Scientific). Disulfide bonds were also confirmed manually.

### Free thiol determination

Free thiols (number of free cysteine residues) were measured in triplicate using Ellman’s reagent (Thermo Scientific) according to manufacturer’s instructions. Pfs230D1+ was diluted (in triplicate) in either ultrapure water or 1.5 M guanidine-HCl to achieve a final concentration of 27 µM prior to conducting the assay. A standard curve was constructed using known concentrations of l-cysteine (3 to 200 µM, Millipore Sigma). Absorbance was measured at 412 nm.

### Generation of mouse anti-Pfs230 antiserum and enzyme-linked immunosorbent assay (ELISA)

CD-1 mice, ten in each group, were immunized with 5.0, 0.5 or 0.05 µg of purified Pfs230C1 or Pfs230D1+ protein formulated with Montanide ISA720 (ISA; Seppic, Fairfield, NJ) adjuvant on day 0 and 21 by intramuscular injection (Noble Life Sciences, Inc., Woodbine, MD). Serum samples were collected on day 42. Due to technical issues, day 42 sera were not collected from one mouse in 0.5 µg of Pfs230C1 group, and five mice in 0.5 µg of Pfs230D1+ group. A second immunization study was conducted using 1 µg of Pfs230C1 or Pfs230D1+ protein formulated with Montanide ISA 720. The immunization studies were conducted under an approved Noble Life Sciences (NLS), the Institutional Animal Care and Use Committee (IACUC) protocol. Noble Life Sciences is an Association for Assessment and Accreditation of Laboratory Animal Care (AAALAC), international accredited, United States Department of Agriculture (USDA) compliant and Office of Laboratory Animal Welfare (OLAW) assured contract research organization.

For the day 42 sera, antibody level against corresponding proteins were determined individually by ELISA. The basic methodology of the ELISA has been described [16] and the minimal detection level of the ELISA was 41 ELISA units to both proteins.

### IgG purification and standard membrane feeding assay (SMFA)

For each group, an equal amount of serum from each mouse was pooled regardless of ELISA units. Total IgGs from the pooled serum sample was purified using Protein G columns (GE Healthcare) according to the manufacturer’s instructions and adjusted to a final concentration of 4 mg/mL in phosphate buffered saline.

The standardized methodology for performing the SMFA has been described previously [17]. Briefly, *P. falciparum* NF54 line was cultured for 16–18 days to induce mature stage V gametocytes. The stage V gametocytes (~ 1% stage V gametocytaemia) were mixed with test IgGs at 750 µg/mL, and the final mixture was immediately fed to ~ 50 female *Anopheles stephensi* through a membrane-feeding apparatus. All feeding experiments were performed with human complement. Mosquitoes were kept for 8 days after feeding and dissected (n = 20 per group) to enumerate the oocysts in the midgut. Only midguts from mosquitoes with any eggs in their ovaries at the time of dissection were analysed. The human serum and red blood cells used for the gametocyte cultures and feeding experiments were purchased from Interstate Blood Bank (Memphis, TN).

### Statistical analysis

To compare ELISA units among three dose groups in each immunogen, Kruskal–Wallis test was utilized, followed by Dunn’s multiple comparisons tests. For SMFA results, the best estimate of  % inhibition in oocyst density [percentage transmission reducing activity (TRA)], and the p-values were calculated using a zero-inflated negative binomial (ZINB) random effects model described previously [18]. All statistical tests were performed in R (version 3.4.1) or Prism 7 (GraphPad).

## Results

### Baculovirus produces a soluble N-terminal domain of Pfs230 (Pfs230D1+)

The new design of a Pfs230 N-terminal construct containing aa 552–731 was based on the previously reported Pfs230C1 TBV antigen (aa 443–731) [10, 14], denoted as Pfs230D1+, and evaluated in the super Sf9/baculovirus system. This design was selected to reduce the potential for proteolytic cleavage and glycosylation while preserving the complete disulfide-linked folding predicted to be required for induction of transmission-blocking antibodies following immunization. Pfs230D1+ also contains an additional N585Q mutation to eliminate potential *N*-glycosylation and a six histidine C-terminal tag to facilitate purification in the initial evaluation here. Moreover, the removal of N-terminal amino acids from the original Pfs230C1 protein did not appear to alter the predicted secondary structure of Pfs230D1+ as suggested by POLYVIEW-2D [19].

**Fig. 1:**
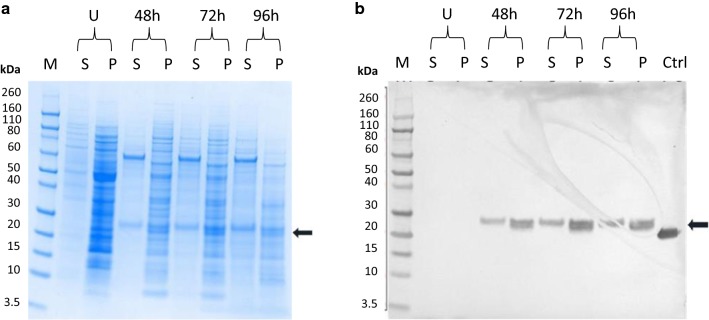
Expression of Pfs230D1+ in super Sf9 cells. **a** Reduced SDS-PAGE and **b** anti-His Western blot analysis of pellets (P) and supernatant (S) samples of Pfs230D1+ in super Sf9 cell (MOI of 1) at 48, 72, and 96 h post infection with uninfected control (U) at 72 h. Molecular weight marker (M) and an irrelevant His-tagged protein used as a positive control for Western blot (Ctrl) are indicated for lane labels. Supernatant (S) fractions show a predominant singular band (and later targeted for purification) while pellet (P) fractions demonstrate a doublet band, both cellular fractions are reactive to anti-His antibodies (**b**)

The expression of Pfs230D1+ was first evaluated with a MOI of 1, 3 or 5 at small-scale (25 mL) and samples were taken at 48, 72 and 96 h post infection to evaluate protein expression. As expression level was independent of MOI, MOI of one was selected for all further analyses. The supernatant and pellet samples were analysed by reducing SDS-PAGE (Fig. 1a) and anti-His Western blot (Fig. 1b) with no evidence of degradation at 96 h post infection. Supernatant fractions of Pfs230D1+ showed a predominant singular band reactive to anti-His antibodies, while pellet fractions demonstrated a doublet also reactive to anti-His but containing a heterogeneous mixture of proteins most likely including misfolded protein (Fig. 1b). Therefore, a MOI of 1 and 96 h of the secreted supernatant fraction was selected for further expression at the 10 L culture scale. Pellet fractions were not targeted for further development and identity of the lower band of the doublet was not interrogated further.

**Fig. 2:**
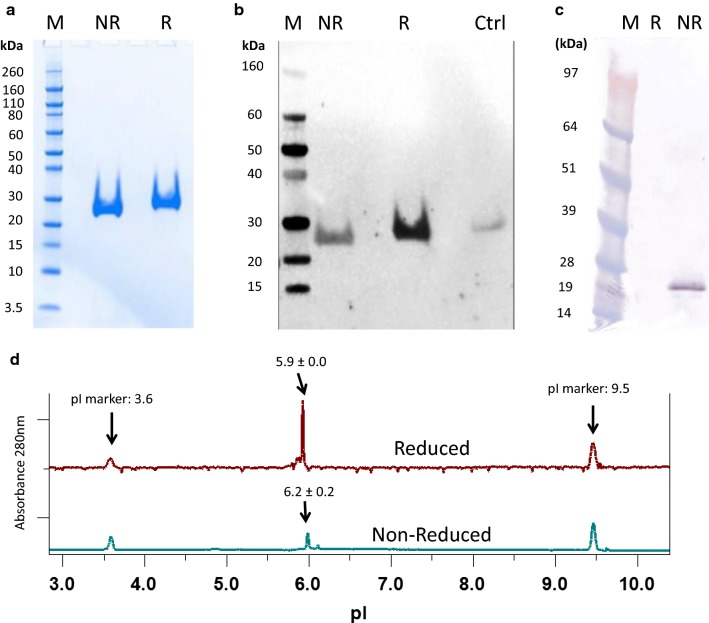
Electrophoresis of purified Pfs230 D1+. **a** SDS-PAGE and **b** anti-His Western blot analysis. **c** Western blot analysis of Pfs230D1+ using mouse monoclonal antibody 15A4-1B12. Lanes are labeled as follows: Pre-stained molecular weight markers (M); non-reduced samples (NR), reduced samples (R), an irrelevant His-tagged protein used as a positive control for Western blot (Ctrl). **d** Representative cIEF electropherograms of Pfs230D1+. A single peak was observed for reduced (~ 5.9) and non-reduced Pfs230DI+ (~ 6.2), which were close to the theoretical pI of 5.6. The reported averages ± 1SD were calculated from three independent experiments

A two-step purification approach was used to capture and polish Pfs230D1+ including IMAC (immobilized metal affinity column) and SEC and resulted in a yield of 23 mg/L as presented here. Further, the resulting purified protein was present at the expected molecular weight of ~ 21 kDa (kilodalton) and with greater than 90% purity by SDS-PAGE and densitometry (Fig. 2a). Reactivity to anti-His antibody confirmed the presence of the histidine tag on the C-terminus (Fig. 2b).

Evidence for the correct disulfide conformation was supported by Western blot using a reduction sensitive conformational monoclonal antibody, 15A4-1B12 [14] as shown in Fig. 2c. In addition, evaluation by capillary isoelectric focusing revealed one major peak in the electropherogram of reduced or non-reduced Pfs230D1+ (Fig. 2d), confirming the presence of a major singular charged isoform. The observed pI of ~ 5.9 for the reduced form and ~ 6.2 for the non-reduced form were close to the theoretical pI of 5.6 for Pfs230D1+ based on the protein’s amino acid sequence.

### Chromatographic analysis of purified D1+

**Fig. 3:**
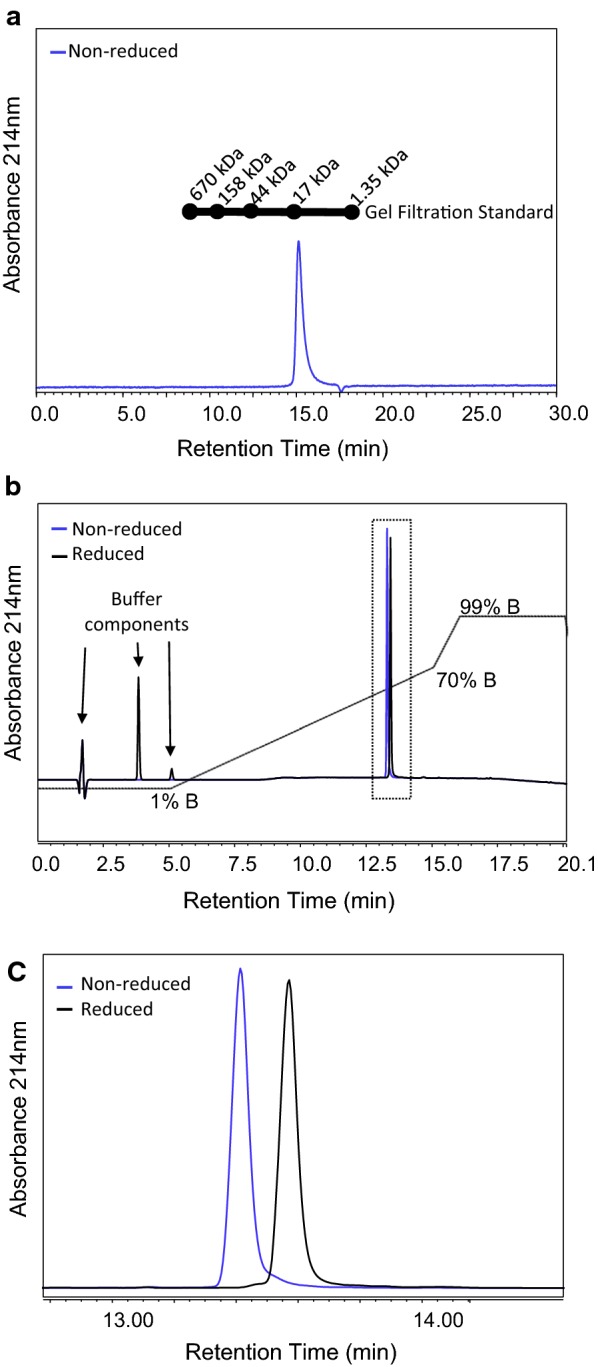
UHPLC analysis of purified Pfs230D1+. Representative **a** SE-UHPLC and **b** RP-UHPLC (reduced and non-reduced) chromatogram of purified Pfs230D1+ with inset zoom (**c**). Blue traces are non-reduced and black traces are reduced samples

Analytical SE-UHPLC was performed to assess the solution state of the purified recombinant protein. Pfs230D1+ eluted as a single peak near the retention time of the 17 kDa SEC standard protein, which was close to the expected size of monomer ~ 21.3 kDa (Fig. 3a). Recovery across the SEC column was ~ 74%; indicating additional higher MW species may be present in the sample. A single peak was also observed by RP-UHPLC under non-reducing or reducing conditions (Fig. 3b, c), indicating the recombinant protein was structurally homogeneous and > 95% pure.

### Intact mass and peptide mapping

**Fig. 4:**
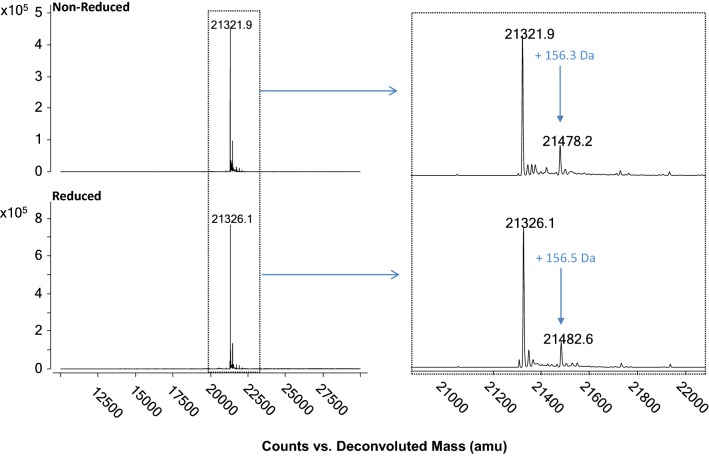
Intact mass analysis of purified Pfs230D1+ (non-reduced and reduced). Representative deconvoluted mass spectra of non-reduced and reduced Pfs230D1+. Inset shows zoom of 21,321.0 Da (non-reduced) and 21,326.1 Da (reduced) corresponding to theoretical masses of 21,321.3 Da and 21,325.3 Da, respectively. Mass additions with second species originating of ~ + 156 Da corresponds to additional N-terminal Arg residue as confirmed by LC/MS peptide mapping (Fig. 5). The reported averages ± 1SD were calculated from three independent experiments

The intact mass analysis indicated that the observed masses of non-reduced (21,321.9 ± 0.0 Da) and reduced (21,326.1 ± 0.0 Da) forms of Pfs230D1+ were similar to their theoretical values of 21,321.3 Da and 21,325.3 Da, respectively (Fig. 4). In addition to the main species, higher mass species were observed under non-reducing (21,478.2 ± 0.0 Da) and reducing (21,482.6 ± 0.1 Da) conditions. These two species are + 156 Da higher than the mass of Pfs230D1+ based on the primary sequence, which corresponded to the presence of an additional Arg residue.

**Fig. 5:**
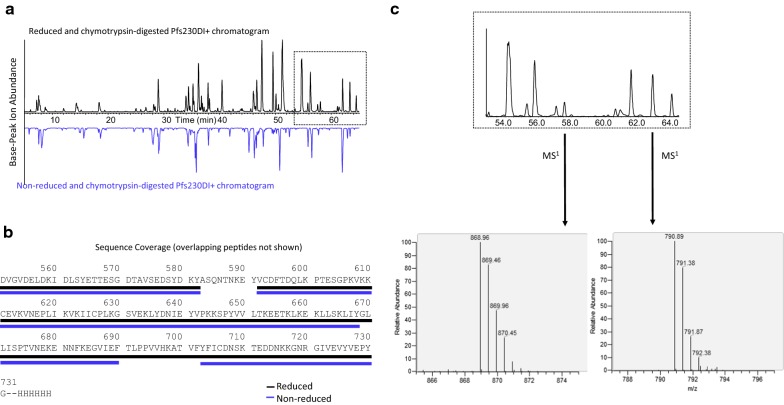
LC/MS peptide mapping of Pfs230D1+. **a** Reduced (top) and non-reduced (bottom) chymotrypsin-digested Pfs230D1+ base-peak ion chromatograms. **b** Sequence coverage of reduced or non-reduced chymotrypsin-digested Pfs230D1+ was 91% and 84%, respectively. **c** In the chymotrypsin-digested Pfs230DI+ base-peak ion chromatogram, peaks at ~ 57.8 min and ~ 63.8 min correspond to the N-terminal peptide with or without an additional Arg residue (+ 156 Da) on the N-terminus, respectively. The relative ion abundance of the N-terminal peptide containing the N-terminal Arg residue was 19 ± 2%, which was determined from six experiments (three independent non-reduced or reduced chymotrypsin digestions)

LC/MS peptide mapping was used to confirm the Pfs230D1+ primary sequence and identify the location of the additional Arg residue (Fig. 5). Primary sequence coverage for non-reduced or reduced chymotrypsin-digested Pfs230D1+ was 84% or 91%, respectively (Fig. 5b). In the chymotrypsin-digested, reduced and alkylated Pfs230D1+ peptide chromatogram (Fig. 5a top), peaks at ~ 57.8 min and ~ 63.8 min were identified as the N-terminal peptide with or without an additional Arg residue (Table 1). The presence of the additional Arg residue was confirmed using MS^1^ (Fig. 5c) and MS^2^ data. The relative ion abundance of the additional Arg residue was 19 ± 2% compared to the N-terminal peptide without the additional Arg. Finally, no glycans were detected by intact mass analysis or LC/MS peptide mapping, indicating Pfs230D1+ was free of glycosylation as a result of the mutation strategy and N-terminal truncation.

**Table 1:** Confirmation of partial N-terminal arginine addition

Peptide	Calculated monoisotopic mass	Observed monoisotopic mass	Mass differences (Da)
D + aa 552–564 DVGVDELDKIDLSY	790.88 Da (MH^+2^)	790.89 Da (MH^+2^)	0.01
N-terminal R + D + aa 552–564 RDVGVDELDKIDLSY	868.94 Da (MH^+2^)	868.96 Da (MH^+2^)	0.02

### Analysis of disulfide bonds

To determine if the four cysteine residues in Pfs230D1+ formed disulfide bonds within the protein, the number of exposed thiol groups was measured using Ellman’s reagent. The results indicated Pfs230D1+ contained < 3% free thiol, under both native and denatured conditions (1.5 M guanidine hydrochloride), which suggested that the four cysteines were likely disulfide paired.

**Fig. 6:**
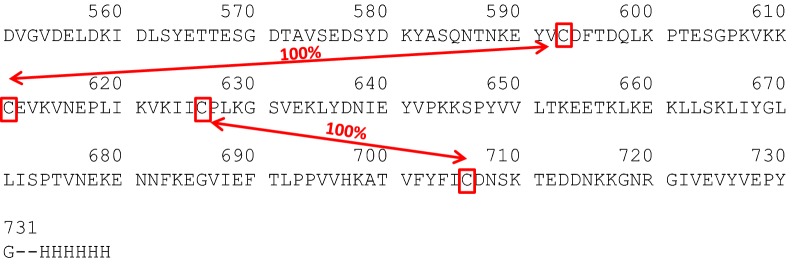
Disulfide pairing of Pfs230D1+ as experimentally confirmed by LC/MS peptide mapping. Pfs230D1+ contains four Cys residues (Cys^593^, Cys^611^, Cys^626^, and Cys^706^). Using the peptides from the non-reduced chymotrypsin digest, disulfide bonds were identified between Cys^593^–Cys^611^ and Cys^626^–Cys^706^. Alternative disulfide bonds or free Cys residues were not observed

Disulfide mapping using LC/MS was employed to determine the disulfide pairing of the four cysteine residues (Cys^593^, Cys^611^, Cys^626^, and Cys^706^) in Pfs230D1+. Using the peptides from the non-reduced chymotrypsin digestion, disulfide bonds were identified between Cys^593^ and Cys^611^ and between Cys^626^ and Cys^706^ (Table 2). Alternative disulfide bonds or free cysteine residues were not observed. A schematic representation of the amino acid sequence of Pfs230D1+ and the elucidated disulfide mapping is provided in Fig. 6. This observed disulfide pairing for Pfs230D1+ matched the predicted bonding pattern of the cysteine-rich motif [6].

**Table 2:** LC/MS analysis of disulfide linked peptides from non-reduced Pfs230D1+

Time	m/z	MS area	Charge	Avg mass	Peptide	Disulfide linkage
50.3	1390.1	5671.8	3	4167.2	A583-L619	Cys^593^ to Cys^611^
46.6	788.4	35,737.6	4	3149.6	V592-L599/K600-L619	
50.8	1566.4	63,264.2	2	3130.5	V592-L619	
60.4	959.4	2012.4	4	3833.7	I620-L628/Y703-Y726	Cys^626^ to Cys^706^
52.8	1175.3	3364.4	3	3522.5	I620-L628/I705-Y726	
58.0	918.5	1964.2	4	3669.9	I620-L628/F704-Y726

### Short-term stability study

**Fig. 7:**
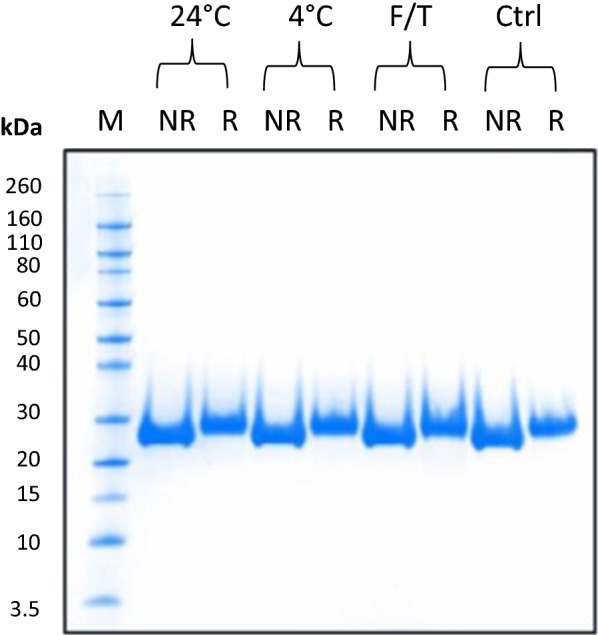
Short-term stability study. Purified Pfs230D1+ was subjected to 24 °C or 4 °C treatment for 7 days or freeze–thaw (F/T) for three cycles at − 70 °C. Samples were run on SDS-PAGE under non-reducing (NR) and reducing conditions (R) alongside untreated control (Ctrl). M: molecular weight marker; 24 °C for 7 days; 4 °C for 7 days; Three freeze/thaw cycles

To support the stability during the various manipulations and analyses reported here, a short-term stability study was conducted for the purified recombinant Pfs230D1+. After storage at 24 °C or 4 °C for 7 days, no sign of aggregation or degradation via SDS-PAGE (Fig. 7) was observed. Further, the Pfs230D1+ protein was evaluated after three additional freeze–thaw cycles, with no changes observed (Fig. 7). These initial studies were designed and developed to support the use and manipulation of Pfs230D1+ during the feasibility and evaluation stage. The stability studies for Pfs230D1+ will be expanded as the preclinical development program continues.

### Immunological evaluation of Pfs230D1+

**Fig. 8:**
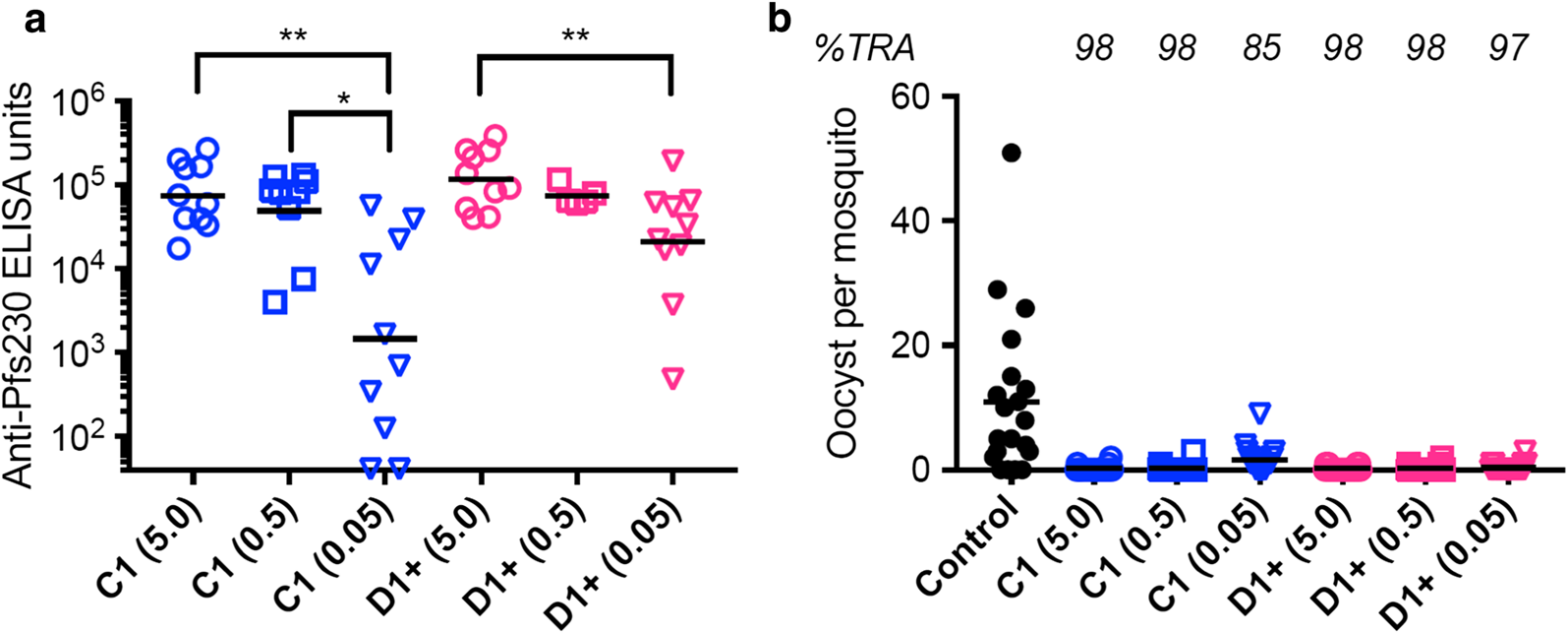
Pfs230D1+ protein induced functional antibodies in mice. **a** Antibody level in each serum was determined against corresponding immunogen by ELISA. Individual (dots) and geometric mean (bars) ELISA units are shown. In each immunogen groups, there was a significant difference among the 3-dose groups (p = 0.001 for Pfs230-C1, and p = 0.015 for Pfs230-D1+ groups by Kruskal–Wallis tests), and significant difference between two groups are shown with asterisks (Dunn’s multiple comparisons test): *p < 0.05; **p < 0.01. **b** Total IgG from each group was tested at 750 µg/mL with complement in SMFA. Oocyst count in individual mosquito (dots) and the arithmetic mean (bars) are shown with % transmission-reducing activity (%TRA) calculated against control group. All inhibitions were significant (p < 0.001 for all by the ZINB model)

To determine whether the Pfs230D1+ protein could induce functional antibodies, as previously shown with Pfs230C1 [10], two independent immunization studies were performed. Study #1 consisted of CD-1 outbred mice immunized with Pfs230C1 or Pfs230D1+ proteins at 5.0, 0.5 or 0.05 µg doses emulsified in Montanide ISA720 adjuvant. When antibody reactivity was assessed by ELISA against the corresponding immunogen (Fig. 8a), both proteins were immunogenic and showed clear dose responses (p = 0.001 for Pfs230C1, and p = 0.015 for Pfs230D1+ groups by Kruskal–Wallis tests). The functionality of antibodies was evaluated by SMFA (Fig. 8b, Table 3). When purified total IgGs were tested at 750 µg/mL in the presence of complement, all IgGs showed significant inhibitions (> 84%TRA, p < 0.001 for all study #1 samples as shown in Table 3). A second immunization study at the 1 µg dose with Montanide 720 for both proteins was then performed to confirm the functional activities observed. Here, in the second study, both anti-Pfs230C1 and anti-Pfs230D1+ purified IgGs also showed significant inhibitions (> 98% TRA, p < 0.001 for both, shown in Table 3). These results demonstrated that Pfs230D1+ recombinant protein could reproducibly elicit biologically active antibodies.

**Table 3:** SMFA results for Pfs230C1 and Pfs230D1+ as tested in two immunization studies

Name	Conc [μg/mL]	Mean oocyst	%TRA	(95% CI)	p-value
Immunization study #1					
Control	750.0	10.9			
C1 (5.0)	750.0	0.2	98.2	(95 to 100)	0.001
C1 (0.5)	750.0	0.2	98.2	(95 to 100)	0.001
C1 (0.05)	750.0	1.7	84.9	(65 to 94)	0.001
D1+ (5.0)	750.0	0.2	98.2	(95 to 100)	0.001
D1+ (0.5)	750.0	0.2	98.2	(95 to 100)	0.001
D1+ (0.05)	750.0	0.4	96.8	(92 to 99)	0.001
Immunization study #2					
Control	750.0	39.2			
C1 (1.0)	750.0	0.5	98.9	(97 to 100)	0.001
D1+ (1.0)	750.0	0.1	99.7	(99 to 100)	0.001

Purified IgG samples were tested at 750 μg/mL by SMFA with complement. Mean oocyst number, % reduction of oocyst density (%TRA), the 95% confidence interval, and p-value of each test IgG are shown

## Discussion

The baculovirus expression system was utilized previously to produce a soluble monomeric N-terminal domain of Pfs230, termed Pfs230C1 (aa 443–731) [10]. It was concluded that this protein was properly folded as it contained the appropriate paired disulfide bonds and produced functional antibodies in mice. However, there was a low level of *O*-glycosylation present which would not be expected in the parasite produced Pfs230 protein. Using peptide mapping and LC/MS/MS, one of the glycosylation sites was identified as serine (aa 551). The present study was aimed to improve the Pfs230C1 construct by further truncating the N-terminal fragment of Pfs230C1 and originating the sequence at valine (aa 552), the amino acid after the *O*-glycosylation site. The intact mass analyses, coupled with the LC/MS peptide mapping indicated that the dual strategy of sequence mutation (N585Q) coupled with the abbreviated N-terminus of Pfs230D1+ (originating at aa 552) eliminated any glycosylation propensity, and resulted in a more pure and more homogeneous molecule than Pfs230C1 [10].

Tachibana et al. produced 30 recombinant Pfs230 fragments across the full-length Pfs230 molecule using wheat germ cell free expression system, five fragments of which elicited transmission-blocking activities and all of them contained cysteine motif 1 (CM 1) [20]. The results from the present study are consistent with this observation [20], as the D1+ fragment (aa 552–731) encompasses the entire functionally active CM1 domain (aa 589–730).

In parallel to the studies conducted here, a shorter Pfs230 fragment (aa 589–731, encompassing the entire CM1 domain alone) was also analysed. This CM1 protein was expressed at substantially lower levels (5 mg/mL) and failed to induce antibodies with functional activity when tested alongside the longer D1+. These results suggest that the 37 amino acids leading to CM1 (present in the D1+ construct) are important for the folding of CM1 domain thereby allowing for better expression/secretion. It should also be noted that Pfs230D1+ is similar to the *Pichia* produced Pfs230D1M (aa 542–736), which is functionally active and encompasses the CM1 domain [7]. The critical traits for Pfs230D1M, such as the overall production yield, the glycosylation profile, as-well-as the presence of potential protease sites in the construct sequence have not been reported [7]. A direct biochemical or biological comparison between Pfs230D1+ and Pfs230D1M was not made in the studies reported here.

Correct disulfide bond formation within recombinant proteins is important to maintain native epitopes and ensure induction of functional antibodies; herein evidence was provided that the appropriate intramolecular disulfide bonds and conformations are present in the Pfs230D1+ protein. A conformational monoclonal antibody, 15A4-1B12 that was previously reported [14] to be reactive to the CM1 domain was used to probe Pfs230D1+. The results indicated positive reactivity in a reduction sensitive manner. Further analysis by peptide mapping with chymotrypsin digests confirmed the correct disulfide bond formation of the four cysteines present in Pfs230D1+.

Approximately 19% of the recombinant Pfs230D1+ contains an additional N-terminal arginine as determined by intact mass analysis and LC/MS peptide mapping. This arginine is not part of the signal peptide from Honeybee Melittin as signal peptide cleavage occurs at the C-terminal Ala^21^ [21, 22]. The same super Sf9 insect cells have previously been used to clone and express other *Plasmodium* antigens [10, 23], resulting in good fidelity; therefore, this is unlikely to be the reason for the arginine addition. Since the protein was expressed via a secretory pathway in insect cells, the inclusion of an N-terminal Asp residue of the signal peptide was not unexpected and has previously been reported [10, 23]; however, Arg addition was not found in the recombinant Pfs25 or Pfs230C1 expressed in baculovirus [10, 23]. As a non-His tagged variant of Pfs230D1+ in the baculovirus system is being developed, additional information about the N-terminal Arg addition may be gained.

The final purified yield for Pfs230D1+ without optimization was 23 mg/L culture, more than twofold higher than achieved for Pfs230C1 [10, 14]. This yield improvement was attributed to, at least in part, the improved stability of the Pfs230D1+ antigen. In the current analysis of Pfs230D1+, the cleavage products were not observed as was seen during optimization of the process for Pfs230C1 [14]. Two of the proteolytic sites identified (via N-terminal sequencing) for Pfs230C1 as C-terminal to Arg^541^ and Gln^545,^ are not present in Pfs230D1+. MacDonald et al. [7] also observed a consistent cleavage C-terminal to Arg^541^ in the *Pichia* expression system when they attempted to express a Pfs230 fragment encompasses aa 444 to 736. Their solution to this issue is similar to that reported here in that Pfs230D1M starts at the amino acid after the cleavage site, Ser^542^ with N585Q mutation to mitigate *N*-glycosylation; however, a second potential protease site (Gln^545^) as well as the potential *O*-glycosylation site (Ser^551^) remains in the Pfs230D1M construct and its glycoprotein profile was not reported. Preliminary, short-term stability studies conducted thus far with Pfs230D1+ (Fig. 7) did not show any degradation or cleaved forms under the conditions tested. Further accelerated studies (including 37 °C) are planned.

## Conclusions

In this study, the theoretical (e.g. glycosylation probability) and empirical data are combined to improve antigen design, resulting in a more stable, homogeneous, and higher expressed TBV candidate that is biologically active. While facilities for baculovirus expression are less common than other systems (e.g. *Escherichia coli* or *Pichia*), some commercial vaccines [24, 25] are being produced using this system, and the data presented indicate it is a viable option for cGMP vaccine manufacturing.

## Data Availability

The datasets used and/or analysed during the current study are available from the corresponding author on reasonable request.

## Abbreviations

ACN: acetonitrile
AAALAC: Association for Assessment and Accreditation of Laboratory Animal Care
BCA: bicinchoninic acid
cIEF: capillary isoelectric focusing
CM 1: cysteine motif 1
CV: column volume
Da: Dalton
DNA: deoxyribonucleic acid
DTT: dithiothreitol
ELISA: enzyme-linked immunosorbent assay
EPA: *Pseudomonas aeruginosa* exoprotein A
HPLC: high performance liquid chromatography
IACUC: Institutional Animal Care and Use Committee
kDa: kilodalton
LDS: lithium dodecyl sulfate
MES: 2-(*N*-morpholino)ethanesulfonic acid
MOI: multiplicity of infection
MS: mass spectrometry
MS^1^: mass spectra^1^
Ni–NTA: nickel–nitrilotriacetic acid
NIAID: National Institute of Allergy and Infectious Diseases
NIH: National Institutes of Health
NLS: Noble Life Sciences
OLAW: Office of Laboratory Animal Welfare
RP: reversed-phase
SDS: sodium dodecyl sulfate
SDS-PAGE: sodium dodecyl sulfate polyacrylamide gel electrophoresis
SE: size exclusion
SEC: size exclusion chromatography
SMFA: standard membrane feeding assay
TBV: transmission-blocking vaccine
TFA: trifluoroacetic acid
TRA: transmission reducing activity
UHPLC: ultra high performance liquid chromatography
USDA: United States Department of Agriculture
ZINB: zero-inflated negative binomial
